# The HDACi Panobinostat Shows Growth Inhibition Both *In Vitro* and in a Bioluminescent Orthotopic Surgical Xenograft Model of Ovarian Cancer

**DOI:** 10.1371/journal.pone.0158208

**Published:** 2016-06-28

**Authors:** Øystein Helland, Mihaela Popa, Katharina Bischof, Bjørn Tore Gjertsen, Emmet McCormack, Line Bjørge

**Affiliations:** 1 Department of Obstetrics and Gynecology, Haukeland University Hospital, Jonas Liesvei 72, 5058 Bergen, Norway; 2 Department of Clinical Science, University of Bergen, PB 7804, 5020 Bergen, Norway; 3 Kin*N* Therapeutics, Laboratoriebygget, Haukeland University Hospital, 5021 Bergen, Norway; 4 Department of Internal Medicine, Haukeland University Hospital, Jonas Lies vei 65, 5021 Bergen, Norway; 5 Centre for Cancer Biomarkers (CCBIO), University of Bergen, 5020 Bergen, Norway; University of Kansas Medical Center, UNITED STATES

## Abstract

**Background:**

In most epithelial ovarian carcinomas (EOC), epigenetic changes are evident, and overexpression of histone deacetylases (HDACs) represents an important manifestation. In this study, we wanted to evaluate the effects of the novel HDAC inhibitor (HDACi) panobinostat, both alone and in combination with carboplatin, on ovarian cancer cell lines and in a murine bioluminescent orthotopic surgical xenograft model for EOC.

**Methods:**

The effects of panobinostat, both alone and in combination with carboplatin, on proliferation and apoptosis in ovarian cancer cell lines, were evaluated using colony and WST-1 assays, Hoechst staining and flow cytometry analysis. In addition, mechanisms were characterised by western blotting and phosphoflow analysis. Immuno-deficient mice were engrafted orthotopically with SKOV-3^luc+^ cells and serial bioluminescence imaging monitored the effects of treatment with panobinostat and/or carboplatin and/or surgery. Survival parameters were also measured.

**Results:**

Panobinostat treatment reduced cell growth and diminished cell viability, as shown by the induced cell cycle arrest and apoptosis in vitro. We observed increased levels of cleaved PARP and caspase-3, downregulation of cdc2 protein kinase, acetylation of H2B and higher pH2AX expression. The combined administration of carboplatin and panobinostat synergistically increased the anti-tumour effects compared to panobinostat or carboplatin treatment alone. In our novel ovarian cancer model, the mice showed significantly higher rates of survival when treated with panobinostat, carboplatin or a combination of both, compared to the controls. Panobinostat was as efficient as carboplatin regarding prolongation of survival. No significant additional effect on survival was observed when surgery was combined with carboplatin/panobinostat treatment.

**Conclusions:**

Panobinostat demonstrates effective in vitro growth inhibition in ovarian cancer cells. The efficacy of panobinostat and carboplatin was equal in the orthotopic EOC model used. We conclude that panobinostat is a promising therapeutic alternative that needs to be further assessed for the treatment of EOC.

## Introduction

Epithelial ovarian cancer (EOC) is the sixth most common malignant neoplasm in women worldwide, and the seventh most common cause of cancer death [1]. Maximum cytoreductive surgery remains the cornerstone in EOC treatment, followed by adjuvant chemotherapy with carboplatin/paclitaxel combination regimens [2]. First line treatment yields a response rate of over 80% and 40–60% complete responses [3, 4]. In the course of the disease, the majority of patients will relapse and develop drug resistance [5]. The overall five-year survival rate is still below 45% [6]. Over the last 10 years, a large number of phase II and III studies have evaluated multidrug combinations, dose-dense scheduling, intraperitoneal delivery routes and maintenance therapy, as well as targeting of angiogenesis and poly/ADP-ribose polymerase (PARP) with marginal or no improvement in overall survival [7–11]. New strategies are therefore to be employed if survival rates are to be improved.

Epithelial ovarian cancer is driven by copy number alterations, somatic mutations and epigenetic changes as acetylation [12]. Epigenetic regulation refers to changes in gene expression by modification of DNA and/or histones, with no alternation of the nucleotide sequence [13, 14]. DNA and histone proteins represent the building blocks of nucleosomes, which are the basic structure of chromatin. These are important for the packaging of eukaryotic DNA, and thereby the control of gene transcription. Acetylation of histones neutralizes the positive charge of the histone tail, and consequently weakens the covalent bindings to the negatively charged DNA, resulting in an open chromatin that facilitates gene transcription [15]. Hyperacetylated histones, therefore, tend to activate genes, and the degree of acetylation is regulated by histone acetyltransferases and histone deacetylase (HDAC)[12]. Hypoacetylation, on the other side can result in downregulation of important tumour suppressor genes such as *Tp53* and *RB1*, can stimulate angiogenesis, and promotes carcinogenesis [16, 17].

HDACs, the important mediator of hypoacetylation, are overexpressed in ovarian cancer tissue [18]. HDACis (Histone deacetylase inhibitors) are a promising class of drugs demonstrating anticancer effects. HDACi can impede cell proliferation and angiogenesis, promote differentiation and induce apoptosis [19]. The effects are mediated both through inhibition of deacetylation of histones and interaction with non-histone proteins such as transcription factors and multiprotein complexes [20, 21]. The HDACis vorinostat and romidepsin, which were approved by the U.S. Food and Drug Administration (FDA) in 2006 and 2009, respectively, are permitted for treatment of T-cell lymphomas [22, 23].

Panobinostat (LBH 589) is a pan-HDACi that has demonstrated more effective antitumour activity against both solid tumours and haematological malignancies than the earliest recognised inhibitors [12]. Panobinostat appears to be the most potent HDACi yet developed; it has been shown to be at least 10 times more potent than vorinostat [12]. Oral panobinostat was accepted by the FDA in 2015 for treatment of patients with recurrent multiple myeloma in combination with bortezomib and dexamethasone [24]. Panobinostat has also demonstrated effective HDAC inhibition in breast, prostate, colon and pancreatic cell lines, while its effects on normal cells were marginal [12, 25]. Although panobinostat has entered phase I/II studies, both with oral and intravenous formulations, for solid tumours, no recruiting studies evaluating the effect of panobinostat on EOC have been identified. Neutropenia, anaemia, thrombocytopenia, hypokalaemia and hypophosphatemia are the toxicities reported, while dose-limiting symptoms may be diarrhoea, nausea and fatigue [14].

In vitro evidence exists for the use of different HDACis (valproic acid, sodium butyrate, vorinostat, belinostat, panobinostat and romidepsin) in EOC, including reports describing inhibited proliferation and induced cell cycle arrest and apoptosis [26–28]. However, only a limited number of studies have investigated the preclinical effects of HDACis in xenograft models [29, 30]. Vorinostat demonstrated a significant increase in survival in an intraperitoneal model of EOC, but only when combined with paclitaxel [31]. No evaluation of HDACis has so far been undertaken in orthotopic models of EOC that permit the study of early, localised disease, tumour cell invasion and dissemination in a biologically relevant order [32].

In this study, we have evaluated the effects of panobinostat, both alone and in combination with carboplatin, on the ovarian cancer cell lines SKOV-3 and CaOv3 in vitro. For the very first time, panobinostat has been assessed in an orthotopic surgical xenograft in vivo EOC mouse model [33]. Panobinostat effectively inhibited growth and induced apoptosis in ovarian cancer cells in vitro, and the effects were synergistic when combined with carboplatin. The drug was well tolerated in the in vivo study. Clinically, panobinostat treatment delayed disease progression and was just as efficient as carboplatin when the survival parameters were analysed.

## Materials and Methods

### Cell Lines and Reagents

The human ovarian adenocarcinoma cell lines SKOV-3 and CaOv3 were obtained from American Type Culture Collection (ATCC Manassas, VA, USA). These cell lines were chosen because of their extensive prior use and characterisation [26, 34]. The cells were cultivated in Dulbecco’s modified Eagle’s medium (DMEM; Gibco, Paisley, UK) and supplemented with 10% heat-inactivated foetal calf serum (FCS; Gibco), 2 mM L-glutamine (Gibco), penicillin 100 IU/ml and 100 μg/ml streptomycin (Gibco) at 37°C in a humidified atmosphere with 5% CO_2_. Cells were grown in 75 cm² cell culture flasks (Costar, Cambridge, MA, USA) and subcultured twice a week. Suspensions of the cells were obtained by washing the cells twice with 10% phosphate buffered saline (PBS; Dulbecco’s tablets, Oxoid Limited, Hampshire, UK) before dissociation of cell monolayers with Trypsin-EDTA (Gibco). Panobinostat was kindly provided by Novartis (Basel, Switzerland), and carboplatin was purchased from Teva (Helsingborg, Sweden). Both drugs were dissolved in DMSO (ATCC^®^ 4-X^™^, U.K.) and stored in aliquots at -20°C until use.

### Colony Formation Assay

SKOV-3 cells (300 cells/well) were seeded in a 6-wells plate (Costar, Cambridge, MA, USA) in DMEM. After 24 hours of incubation, the media was changed and the cells were treated with 0, 3.1, 6.2, 12.5, 25 or 50 nM panobinostat. After 72 hours, the cells were gently washed and allowed to form colonies in the complete medium without drugs for another 10 days, before being fixed with 95% methanol, washed with PBS and stained with a tryphan blue stain solution (Thermo Fischer Scientific, Hannover Park IL, USA). Colonies comprising more than 50 cells were enumerated.

### Annexin V-FITC/Propidium Iodide (PI) Flow Cytometry

SKOV-3 and CaOv3 cells were seeded in DMEM in 6-well plates (Costar, Cambridge, MA, USA) with 2 x 10^5^ cells in each well. Cells were incubated for 24 hours; the media was then changed, and the cells were treated with 0, 3.1, 6.2, 12.5, 25 or 50 nM of panobinostat. After 72 hours, the cells were collected and concentrated by centrifugation. Cells were re-suspended, and cells in different apoptotic stages were identified using the Muse Annexin V & Dead Cell Assay Kit (Merck Millipore, Billerica, MA, USA). The assay was carried out as described by the manufacturer’s protocol. The cell sample was analysed by use of the Muse^TM^ Cell Analyzer (Merck Millipore).

### Morphological Changes Observed by Hoechst 33342 Staining

Percentages of abnormal/apoptotic SKOV-3 cells were determined after fixation and staining with 4% paraformaldehyde, supplemented with 10 μg of the DNA-dye, bisbenzimide (Hoechst 33342, Calbiochem, San Diego, CA, USA). The different treatment regimens were: panobinostat (0, 0.062, 0.310, 0.621, 3.10, 6.21, 31.0, 62.1, 310, 621 and 3100 nM), carboplatin (0, 0.175, 0.35, 1.75, 3.5, 17.5, 35, 175, 350, 1750 μM) and panobinostat/carboplatin combined. 300–500 cells were counted for each well. Viable cells had a uniform diffused and well-defined nuclear fluorescence, while the apoptotic cells appeared condensed and/or fragmented, with more intense staining [35]. The morphology was analysed using a Leica DM IRB epifluorescence microscope (Leica, Bensheim, Germany).

### Cell Cycle Analysis

SKOV-3 cells were seeded into 6-well plates (2 x 10^5^ cells per well) for 24 hours before being treated with 0, 3.1, 6.2, 12.5, 25 or 50 nM of panobinostat for 48 hours. Suspensions of cells were obtained as described above; the cells were then fixed in ice-cold methanol (Kemetyl, Vestby, Norway). The cells were re-suspended in PBS, and the Muse Cell Cycle Kit (Merck Millipore, Billerica, MA, USA) was used to measure the cellular DNA content, according to the manufacturer’s instructions. The Muse Cell Analyzer (Merck Millipore) was used for the quantitative measurements, and the number of cells was plotted as a function of DNA content.

### Analysis of Cell Viability

Cell viability was determined using the cell proliferation reagent WST-1 (Roche Applied Science). Aliquots (100 μl) of SKOV-3 and CaOv3 (50,000 cells/ml) were seeded into 96 well-plates (Costar), and cultured with the following concentrations of panobinostat (0, 0.062, 0.310, 0.621, 3.10, 6.21, 31.0, 62.1, 310, 621 and 3100 nM), carboplatin (0, 0.175, 0.35, 1.75, 3.5, 17.5, 35, 175, 350 and 1750 μM), or a combination of these concentrations. After 72 hours, 10 μl WST-1 was added for two hours before the absorbance at 620 nm was measured with a microplate reader (Tecan Infinite 200, software Magellan version 6). The results are presented as a ratio of treated viable cells relative to viable control cells, using the formula: [(treated viable cells) / (control viable cells)] x 100. The effect of combining panobinostat with carboplatin was calculated [36].

### Western Blot Analysis

SKOV-3 cells were plated at 75% confluence in 6-well plates, and allowed to attach overnight. Cells were treated with panobinostat at 0, 3.1, 6.2, 12.5, 25 or 50 nM for 24 hours before being lysed with a cell lysis (Shieh) buffer. Protein concentrations were determined, and equal amounts of protein were subjected to SDS-polyacrylamide gel electrophoresis and electroblotted onto nitrocellulose membranes. After blocking with 5% non-fat skimmed milk in tris-buffered saline with tween (TBST) for one hour at room temperature, the blots were incubated with primary antibodies diluted in a blocking buffer and directed against p21 (ab16767, Abcam, Cambridge, UK), PARP-1 (Santa Cruz Biotechnology Inc., Santa Cruz, CA, USA), H2B (Upstate, Lake Placid, USA), caspase-3 (sc-7148, Santa Cruz, CA, USA), cdc2 (sc-166135, Santa Cruz, CA, USA), α-Tubulin (mAb #3873, Sigma, Saint Louis, USA), cox IV (ab183629, Abcam) and GAPDH (mAbcam 9484, Abcam) for one hour at 37 C. GAPDH, cox IV and tubulin were used as loading controls. After washing and incubation with appropriate horseradish peroxidase conjugated secondary antibodies (Jackson ImmunoResearch, West Grove, PA, USA), the bound antibodies were detected with the use of the Pico Stable peroxide solution and luminol enhancer solution (Pierce Biotechnology, Inc., Rockford, IL, USA). KodakImage Station 4000R (Eastman Kodak Company, Rochester, NY, USA) was used for visualisation.

### The DNA Damage Mark pH2AX Detection

To examine whether the cells were injured by the DNA damage response pathway, the level of H2AX phosphorylation (pH2AX) was measured. SKOV-3 cells were seeded into 6-well plates (2 x 10^5^ cells per well) for 24 hours before being treated with 0, 25 or 50 nM of panobinostat for 24 hours. To measure the DNA damage mark from gamma-H2AX phosphorylation (pH2AX), the MuseTM H2AX activation detection kit (Merck Millipore, Billerica, MA, USA) was performed according to the manufacturer’s description.

### Retroviral Transfection of SKOV-3 Cells

Retroviral transfection with the establishment of SKOV-3 clones, which steadily expressed luciferase denoted as SKOV-3^luc+^, was performed as described earlier [33].

### Animals

The protocol for animal studies was approved by the Norwegian State Commission for Laboratory Animals (ID 4602), and the experiments were performed according to the European Convention for the Protection of Vertebrates Used for Scientific Purposes. Female NSG mice (six to eight weeks old; Vivarium, University of Bergen) were maintained under defined flora conditions in individually ventilated (HEPA-filtered air) sterile micro-isolator cages (Techniplast, Buguggiate, Italy) at the University of Bergen’s animal facility. No more than five mice were placed in each individually ventilated cage; cages were kept on a 12-hour dark/night schedule at a constant temperature of 21°C, and at 50% relative humidity. Bedding and cages were autoclaved and changed twice per month. The mice were offered a continuous supply of sterile water and food, were monitored daily by the same staff for the duration of the experiment, and were weighed three times per week. During imaging, mice were anaesthetised with 3% isoflurane (Isoba Vet, Schering-Plough, Brussels, Belgium).

### Toxicology Study

In this study, all drugs and control substances were administered through the intraperitoneal route. In human studies, intraperitoneal delivery has shown at least the same response rate as the intravenous route [37, 38]. To determine the maximum tolerated dose (MTD) of panobinostat, the following dosages and schedules were evaluated: panobinostat at 15, 25 or 50 mg/kg, five days per week, for three consecutive weeks (Q5Wx3), with three mice in each group. Panobinostat was dissolved in 10% DMSO. The controls were matched for the concentration of the solvent. Carboplatin was tested in an earlier study [33]. Based on the combined results, an amalgamation consisting of panobinostat at 7.5 mg/kg (Q5Wx3) and carboplatin at 12 mg/kg (Q2Wx3) was chosen for further studies. During the MTD study, body weight was monitored daily for 28 days, and at the end of the study the mice were euthanized.

### Necropsy

The health status and weight of the mice were monitored daily, and mice were humanely euthanized when moribund, as defined by weight loss of > 10–15% of body weight, signs of lethargy or ruffled fur.

### Orthotopic Ovarian Cancer Model

The establishment of orthotopic xenografts was performed as described earlier [33]. Formation and growth of tumours was followed by bioluminescent imaging (BLI). Surgical intervention was performed 21 ± 7 days after orthotopic injection of tumour cells. Prior to initiation of the therapeutic study, mice were divided into groups based on BLI and body weight. We performed an ANOVA analysis to avoid selection bias. The surgical procedure and optical imaging technique used, as well as the administration methodology of chemotherapeutics, were the same as described earlier [33].

### Design of Trials

The therapeutic schedules were divided into an isolated chemotherapeutic cohort and a combined surgical/chemotherapeutic cohort. The mice were randomised into four different treatment arms in both cohorts, with an equal number of mice in each group (*n* = 6). First cohort (chemotherapeutic): (a) control, (b) panobinostat 15 mg/kg (Q5Wx3), (c) carboplatin 20 mg/kg (Q2Wx3) and (d) carboplatin 12 mg/kg (Q2Wx3) + panobinostat 7.5 mg/kg (Q5Wx3). Second cohort (surgical/chemotherapeutic): (a) control, (b) surgery alone (hysterectomy, bilateral salpingo-oophorectomy and removal of metastasis if present), (c) panobinostat 7.5 mg/kg (Q5Wx3) + carboplatin 12 mg/kg (Q2Wx3) and (d) surgery, followed by panobinostat 7.5 mg/kg (Q5Wx3) + carboplatin 12 mg/kg (Q2Wx3). Efficacy was evaluated throughout the study period using BLI and survival time.

### Statistical Methods

Cell analysis was based upon triplicates, and results are given as means +/- standard deviation. The statistical significance of differences between treatment groups in vitro and in vivo was determined using a two-tailed student *t* test. Synergism was calculated by bliss independence analysis [36]. Survival data was analysed using the Kaplan and Meier method. The Mantel-Haenzel log-rank statistics (GraphPad Prism 5.0, GraphPad Software, La Jolla, CA) was used to analyse survival distribution.

## Results

### *In Vitro* Effects of Panobinostat

**Fig 1:**
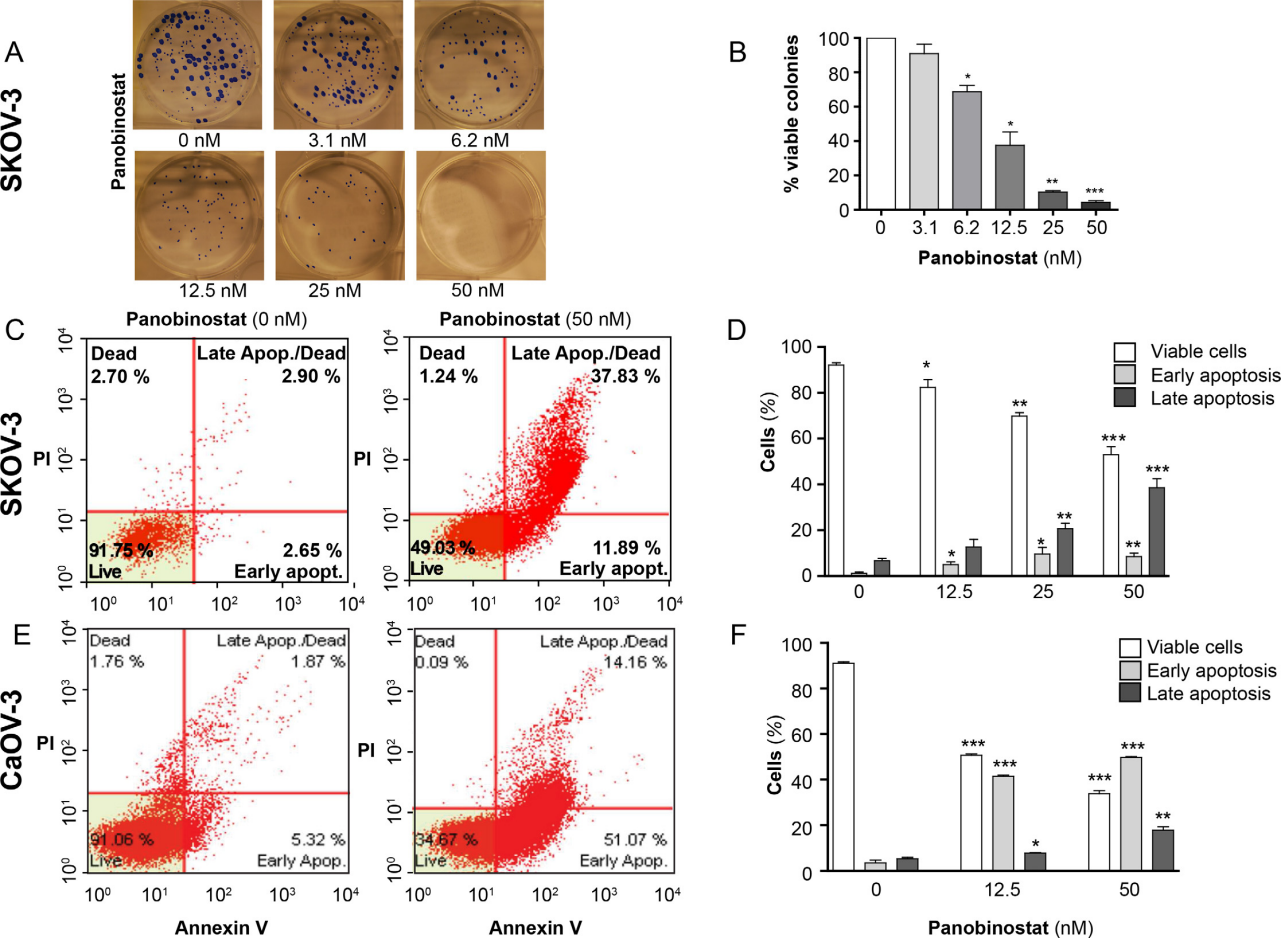
Panobinostat inhibits colony formation and induces cell death of SKOV-3 cells assessed by flow cytometry after 72 hours of treatment. **A:** Representative images of the colony formation after exposure to various drug concentrations. The blue dots represent vital cell clusters. **B:** Percentages of viable colonies relative to control (untreated) cells. **C:** Apoptosis for SKOV-3 measured by annexin V/PI staining. Viable cells (lower left quadrant) were negative for both annexin V and PI, apoptotic cells were positive for annexin V staining and negative for PI, and the dead/late apoptosis cells were positive for both annexin V and PI (upper right quadrant). **D:** Percentages of the different cell death stages for SKOV-3. All results were presented as mean ± SD (*n* = 3), * *P* < 0.05, ** *P* < 0.005 and *** *P* < 0.001 compared to controls, calculated by unpaired t-test. **E, F**: Apoptosis by annexin V/PI staining and percentage of the different cell death stages for CaOv3.

A colony formation efficacy assay was performed to assess the effects of panobinostat on SKOV-3 cell proliferation (Fig 1A). The results, which are summarised in Fig 1A and 1B, show a dose-dependent inhibition. Already, after exposure to 3.1 nM panobinostat, a reduction of visible colonies could be demonstrated. A significant reduction of colonies was seen after 6.2 nM (*P* = 0.013). After treatment with 25 nM, almost no colonies of SKOV-3 were identified (*P* < 0.0001). No visible colonies were detected when the cells were treated with 50 nM of panobinostat.

When SKOV-3 and CaOv3 cells were exposed to 12.5 nM of panobinostat for 72 hours, a significant increase in cell death was observed by use of annexin V/PI staining flow cytometry. Different stages of apoptosis (early and late apoptosis, and necrosis) were identified, a significant increase of all stages (*P* < 0.05) was observed, and the effect was increased in a dose-dependent manner (Fig 1C–1F). In addition, a significant increase of cells entering late apoptosis was observed when the cells were exposed to 50 nM (Fig 1D–1F).

**Fig 2:**
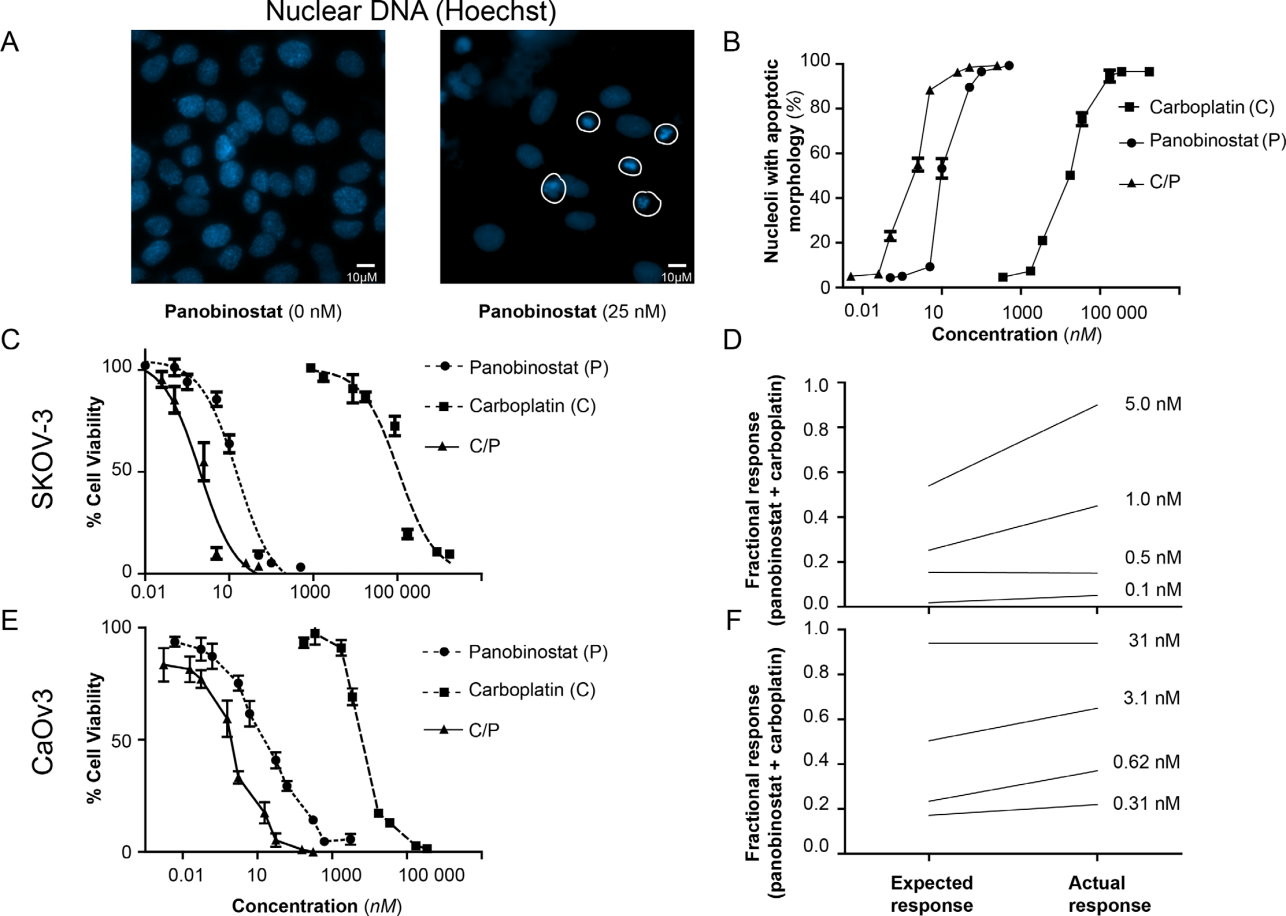
Apoptosis on SKOV-3 cells after treatment with panobinostat, carboplatin or a combination of these drugs for 72 hours. **The combination of panobinostat and carboplatin resulted in synergism. A:** Nuclear morphology was evaluated by Hoechst 33342 staining. Abnormal nuclei with condensed chromatin were consistent with cell apoptosis/death (highlighted in white). **B:** The percentages of apoptotic nuclei are shown as mean ± SD (*n* = 3). **C:** Cell viability for SKOV-3 is measured with the use of the WST-1 assay. **D:** The effect of combining panobinostat and carboplatin on SKOV-3 was calculated from the response to each of the drugs alone, compared to the expected response by combining similar concentrations (calculated by Bliss independence analysis). A positive difference between actual response and expected was then ascribed to synergy. **E,F:** Cell viability for CaOv3, and Bliss independence analysis of expected and actual responses for combinational therapy of panobinostat and carboplatin for CaOv3.

Morphological changes, due to cellular apoptosis and cell death, were seen after exposure to panobinostat, carboplatin or a combination of both. By the use of a fluorescence microscopy, nuclear changes in cells were detected with Hoechst staining (Fig 2A). Treatment with both panobinostat and carboplatin showed a dose-dependent effect, while a potentiated effect was seen when combinations of the two were used (Fig 2B).

The WST-1 assay was used to assess cell viability after exposure to panobinostat, carboplatin or a combination of these drugs for 72 hours. Panobinostat monotherapy resulted in a significant reduction in viability, with a half-maximal inhibitory concentration (IC50) of 15 nM. IC50 for carboplatin was 69 μM. When panobinostat and carboplatin were combined, the IC50 value was calculated to be 1 nM for panobinostat (Fig 2C). Analysis of the combination treatment demonstrated a higher actual response than expected for the specified concentrations (Fig 2D and 2F), reflecting a synergistic effect. The effect is, however, not as noticeable for CaOv3 as for SKOV-3.

**Fig 3:**
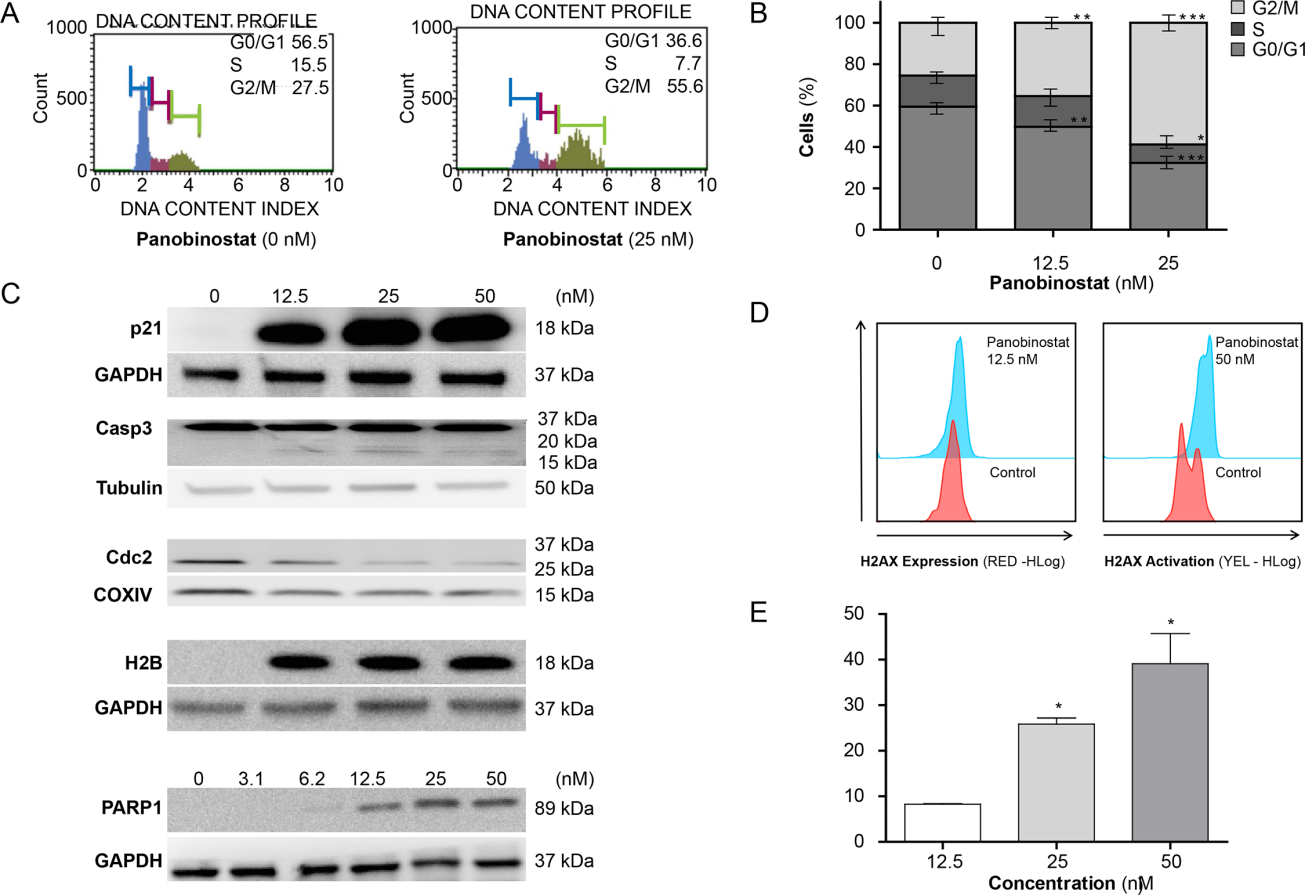
Exposure of SKOV-3 cells to panobinostat for 24 hours results in cell cycle arrest and upregulation of proteins regulating cell cycle arrest, histone acetylation and cell death. **A, B:** Cell cycle arrest revealed by cell cycle analysis. **C:** Upregulation of p21 and H2B. PARP1 and caspase-3 cleavage and activation of cdc2 detected by immunoblotting techniques. **D, E:** Increased phosphorylation of H2AX examined by phosphoflow cytometry.

To explore if the inhibition of ovarian cell line proliferation by panobinostat was due to cell cycle arrest, we analysed cell cycle distribution using flow cytometry (PI staining). Panobinostat treatment altered cell cycle scatter in the SKOV-3 cells, and resulted in an accumulation of the cells in the G2/M phase and a significant decrease of cells in both the S (*P* < 0.05) and G0/G1 (*P* < 0.01) phases (Fig 3A and 3B). The exposure also resulted in a dose-dependent increase in the sub-G/debris fraction (data not shown).

By immunoblotting, panobinostat was found to induce hyper-acetylation of histone H2B protein (Fig 3C). Protein levels of p21 and the apoptotic cleavage products of PARP-1 were both upregulated in a dose-dependent manner. The cell-regulatory protein, cdc2, was downregulated and caspase-3 was cleaved. When examining the apoptosis regulators Bak, Mcl-1 and Bcl-2, and the cell-regulatory protein cyclin D1 no consistent pattern was revealed (data not shown). The loading control GAPDH and tubulin showed consistent expression (Fig 3C). After SKOV-3 cells were treated with panobinostat for 24 hours, a dose-dependent induction of pH2AX was seen (Fig 3D and 3E).

### Chemotherapy and Surgery in a Bioluminescent and Orthotopic Xenograft Model

The MTD studies identified carboplatin at 20 mg/kg [39], panobinostat at 15 mg/kg and a combination of carboplatin at 12 mg/kg and panobinostat at 7.5 mg/kg to be well tolerated, with no significant loss of body weight (S1 Fig). No major complications of surgery were to be reported. Except for the first three days after the surgical procedure, all mice gained weight over the following three weeks. When their performance status declined due to progression of the disease, they started to lose weight again (S2 Fig).

**Fig 4:**
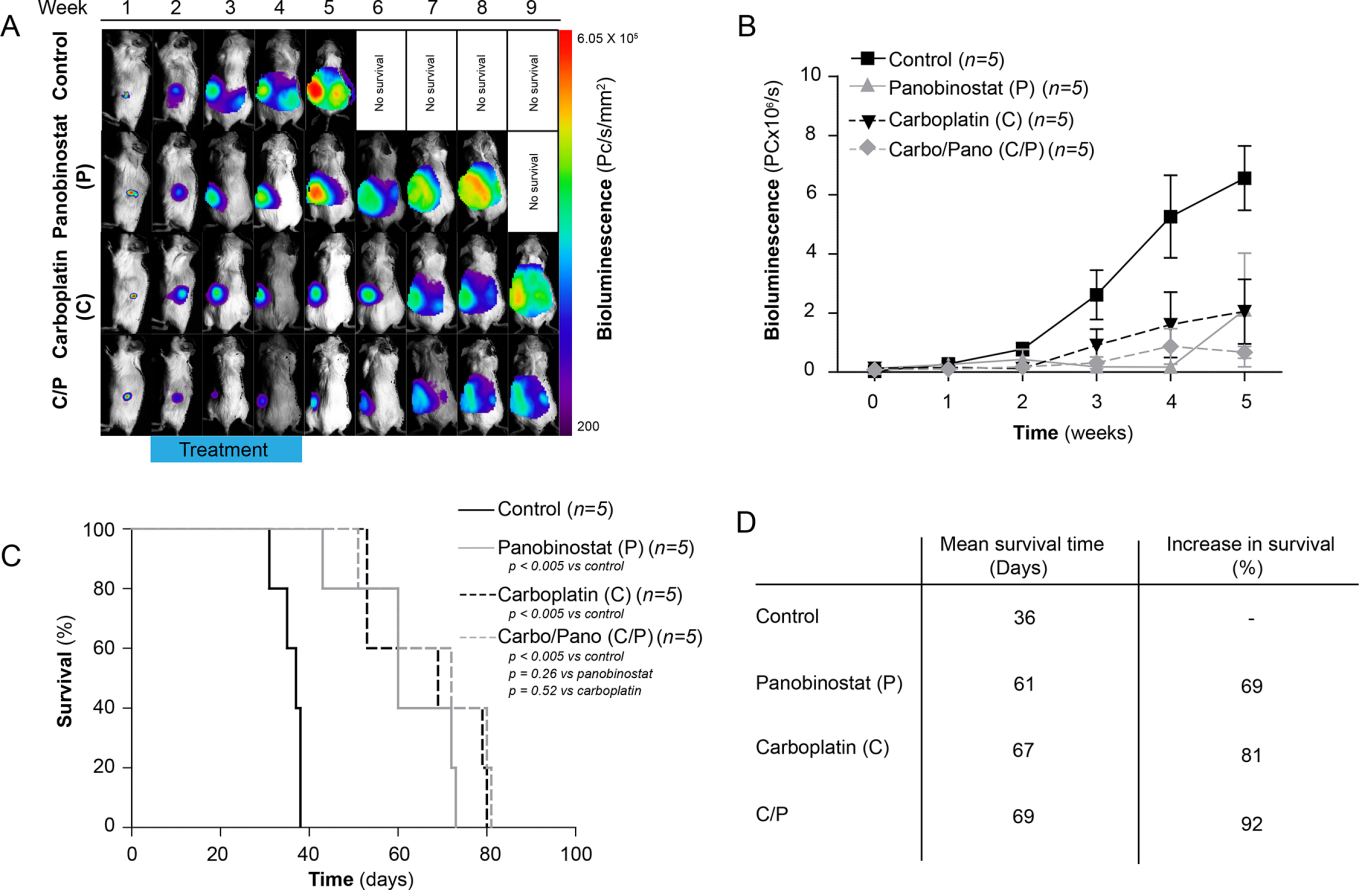
Chemotherapeutic in vivo cohorts. **A:** Illustration of weekly bioluminescent image analysis of representative groups of xenografted mice: a) control, b) panobinostat, c) carboplatin and d) combination of panobinostat and carboplatin. **B:** Relative tumour growth measured by BLI. **C:** Kaplan-Meyer cumulative survival curves of control, panobinostat-, carboplatin- and a combination of panobinostat- and carboplatin-treated mice. **D:** Median survival time and increase in survival time (%) for the variously treated groups.

Panobinostat and carboplatin monotherapies significantly increased the mean overall survival to 69% (*P* < 0.005) and 81% (*P* < 0.005), respectively, compared to the control group. The difference between the two treatment groups was not significant (*P* = 0.85). A 92% increase in survival time was observed when carboplatin and panobinostat were combined (*P* < 0.005), compared to the control group, but the effect was not significant when compared to single agent panobinostat (*P* = 0.26) or carboplatin (*P* = 0.52) treatment (Fig 4C and 4D).

**Fig 5:**
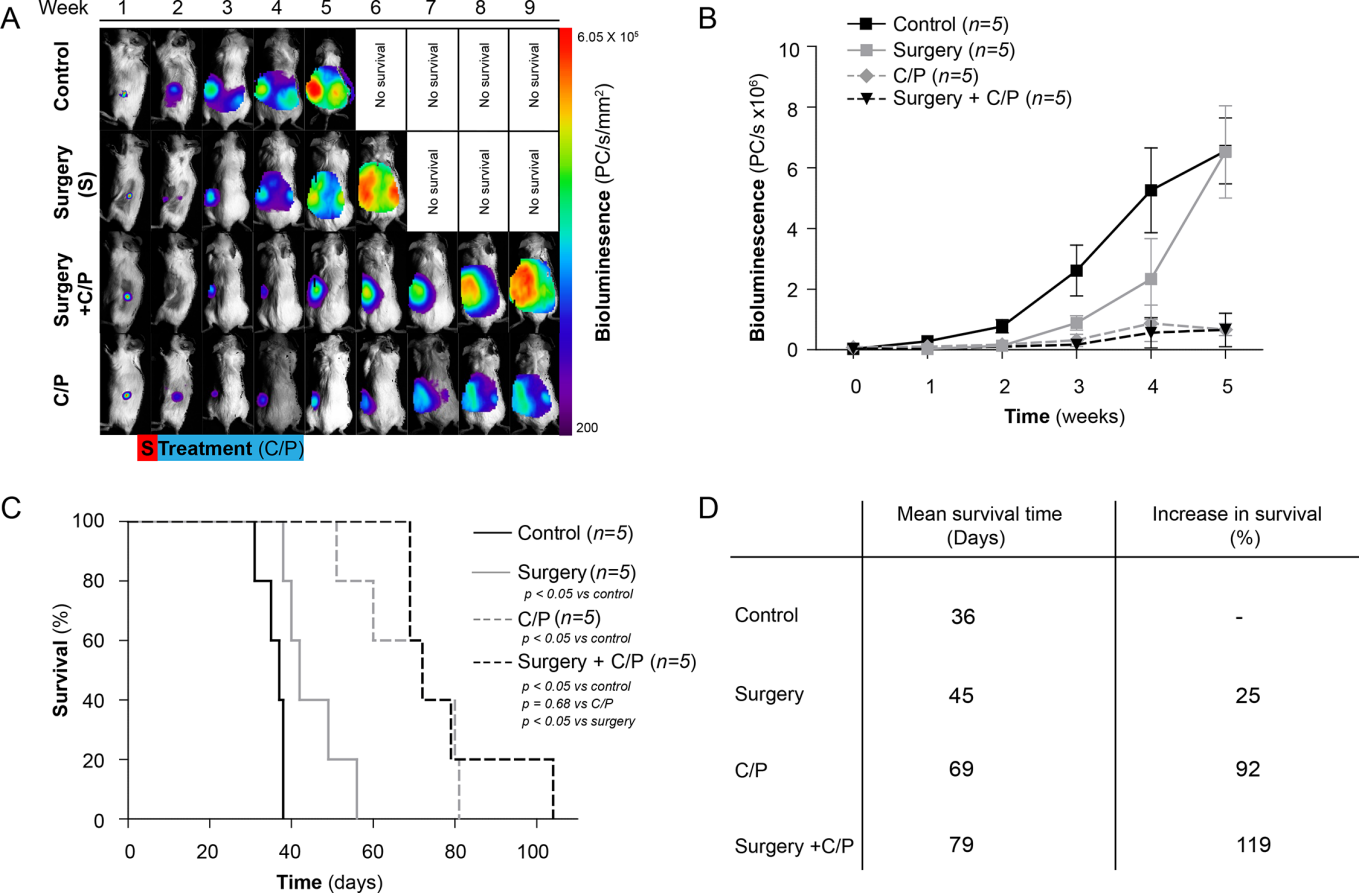
Surgical in vivo cohorts. **A:** Illustration of weekly bioluminescent image analysis of representative group of xenografted mice: a) control, b) surgery, c) surgical and panobinostat/carboplatin-treated and d) combination of panobinostat and carboplatin. **B:** Relative tumour growth measured by BLI. **C:** Kaplan-Meyer cumulative survival curves of control, panobinostat-, carboplatin- and a combination of panobinostat- and carboplatin-treated mice. **D:** Median survival time and increase in survival time (%) for the variously treated groups.

A combined surgical/chemotherapeutic xenograft model of EOC has recently been established [39], and the effects of surgery, panobinostat/carboplatin, and surgery followed by panobinostat/carboplatin were analysed. An increase in the BLI signal corresponded to progress of the disease and overall survival (Fig 4A and 4B). The surgical cohort with no post-operative treatment had a final 25% increase in survival compared to the control group (*P* < 0.05) (Fig 5). The differences in mean overall survival for the carboplatin/panobinostat (68.8 ± 3.4 days) and surgery + carboplatin/panobinostat cohort (78.6 ± 3.6 days) were, however, not significant (*P* = 0.65) (Fig 5C and 5D). Further, through the autopsies differences in the pattern of metastatic spread between the treatment and control groups could not be revealed.

## Discussion

In the present study, we have shown that HDACi panobinostat inhibited growth and induced apoptosis in EOC cells in vitro. In addition, we demonstrated significant prolongation of survival time by administration of panobinostat, carboplatin or panobinostat/carboplatin in an orthotopic EOC xenograft model. Panobinostat was equally as effective in extending survival time as carboplatin. No additional effect on survival parameters was seen when panobinostat was combined with carboplatin, and when surgery was combined with carboplatin/panobinostat treatment.

There are a limited number of studies assessing the effects of panobinostat on ovarian carcinoma cells in vitro. The inhibition of cell proliferation and induction of apoptosis, shown in our report, are in accordance with these previous findings [26, 34] (Figs 1–3). Initiation of cell demise was shown by finding increasing amounts of PARP cleavage products and cleavage of caspase-3 (Fig 3C), while panobinostat-mediated acetylation of H2B (Fig 3C) and up-regulation of pH2AX (Fig 3D and 3E) suggest that the effects are mediated by HDAC inhibition [40]. Although described for other carcinomas [41], the anti-proliferative effects of panobinostat through cell cycle arrest had not previously been described for ovarian carcinoma cells (Fig 3A and 3B). We suggest, in line with what is described for pancreatic cancer treated with the HDACi belinostat [42], that the halt of cells in the G2/M phase to be, at least in part, mediated by an interaction with p21. The latter was induced in a dose-dependent manner upon downregulation of the cdc2 protein kinase (Fig 3C).

In our in vitro studies, concentrations were chosen to cover the entire spectrum of clinically achievable free panobinostat concentrations [43]. Compared to other HDACis, panobinostat is very effective [40], and the IC50 values for ovarian cancer cell viability confirm this (Fig 2C and 2E. The efficacy was even more potent than for treatment with carboplatin alone. A preclinical test system, which allowed analysis of synergism or antagonism of up to three different agents in defined cell lines in vitro, showed similar results [34]. In addition, panobinostat combined with carboplatin and gemcitabine has demonstrated synergistic effects [34]. The synergistic activity of panobinostat when used together with other cytostatics is of interest, as combinations of agents are widely used in the treatment of EOC in an attempt to achieve a synergistic therapeutic effect, dose and toxicity reduction, and the minimisation or delay of the induction of drug resistance.

Panobinostat treatment of the orthotopically xenografted mice suppressed tumour growth, and significantly increased the mean overall survival (Fig 4). The potency was similar to what was identified for carboplatin, the main pillar of all primary treatment regimens [44], suggesting that this represents a potential alternative for patients that cannot be treated with carboplatin [45]. Treatment alternatives are needed for those patients who have experienced severe allergies or other toxic side effects [46], as well as for those who have developed drug resistance. As shown in the toxicology study, treatment with panobinostat alone, and when combined with carboplatin, was reasonably well tolerated by the mice (S2 Fig). Allergic reactions that are known to inhibit clinical use of different cytostatic drugs [47] have not, to our knowledge, been reported for panobinostat. Together with the described acceptable safety and tolerability profile in humans, this makes panobinostat a promising substance to be examined further in clinical human EOC studies [48]. Although we have used an intraperitoneal administration form, panobinostat is known to have favourable pharmacokinetic characteristics, thus making oral administration of this drug an attractive alternative [24, 49].

More than 70% of patients with EOC will need second-line chemotherapy, due to recurrence of the disease within 18 months. The response rates to the treatment alternatives decreases with each subsequent line of therapy [5], and many patients will develop a resistance to carboplatin [50]. The use of alternative treatment options to carboplatin regimen, in order to prolong the platinum-free interval, has gained increased interest as it may augment the likelihood of response to platinum if reintroduced at later relapses [51–53]. It is therefore important to determine whether panobinostat can represent a non-platinum treatment alternative for patients with a partially platinum-sensitive disease, who will eventually benefit from a delay in platinum re-treatment [54, 55].

Discrepancies between in vitro and in vivo results, as well as between xenograft models and clinical trials, have been shown previously [56, 57]. This may be explained by the differences in complexity between the model systems, and could be driven by the less effective exposure of the respective agents. We were not able to follow up the promising in vitro results when carboplatin and panobinostat were combined. However, few HDACis have been tested in in vivo models [58] and this is the very first in vivo study of panobinostat against ovarian cancer.

In contradiction to the findings shown when the mice were treated with carboplatin/paclitaxel and surgery [33], no additional effect on survival parameters was seen when surgery was combined with panobinostat/carboplatin, compared to panobinostat/carboplatin treatment alone (Fig 5). The mice in the present cohorts were allowed to develop a more advanced stage of disease than in our earlier study [33] before treatment was initiated, and sufficient debulking was not achieved (Fig 5). The limited success of the combination therapy is therefore, at least in part, the presence of increased micrometastasis not visible to the surgical team and not merely due to the use of the alternative chemotherapeutic regimen.

## Conclusion

New strategies must be employed if survival rates are to be improved for ovarian cancer. Alternative options to standard carboplatin chemotherapeutic regimen are necessary for when carboplatin cannot be utilised. This is the first study to show the potential benefits of the use of the HDACi panobinostat in an orthotopic xenograft model of ovarian cancer. These results, together with the data from human in vitro studies, suggest that panobinostat should be evaluated in clinical human EOC trials.

## Supporting Information

S1 Fig Toxicology study with measurements of weights after treatment with panobinostat, carboplatin or a combination of these drugs. Percentage changes in weights for the mice after (A) carboplatin, (B) panobinostat and (C) a combination of panobinostat and carboplatin.
(EPS)

S2 Fig Surgery and a combined treatment with panobinostat and carboplatin affect the weights. Percentage changes in weights for surgical group and combination group (surgery followed by panobinostat and carboplatin).
(EPS)
